# A note on the distance distribution paradigm for Mosaab-metric to process segmented genomes of influenza virus

**DOI:** 10.5808/GI.2020.18.1.e7

**Published:** 2020-03-31

**Authors:** Mosaab Daoud

**Affiliations:** Independent Research Scientist, Toronto, ON M1S1B2, Canada

**Keywords:** composite data point, distance distribution paradigm, Mosaab-metric space, segmented genome of influenza virus

## Abstract

In this paper, we present few technical notes about the distance distribution paradigm for Mosaab-metric using 1, 2, and 3 grams feature extraction techniques to analyze composite data points in high dimensional feature spaces. This technical analysis will help the specialist in bioinformatics and biotechnology to deeply explore the biodiversity of influenza virus genome as a composite data point. Various technical examples are presented in this paper, in addition, the integrated statistical learning pipeline to process segmented genomes of influenza virus is illustrated as sequential-parallel computational pipeline.

## Introduction

One of the main advances in bioinformatics, computational biology, and biotechnology is the sequence-set analysis. It is a new research direction parallel to sequence analysis. The main idea behind it is to analyze composite data points in data space, feature space or distance space. A composite data points is a dataset, for example set of feature vectors, set of sequences. This generalized concept proposed in [1,2]. Now, dealing with segmented genomes of influenza virus as composite data points has different aspects: biodiversity, bio-intelligent system, genomic variation, and vaccine efficiency.

The influenza viruses have a negative impact on public health and still creating threats for different life aspects. The early pandemic of H1N1 flu virus started in 1918 [3]. Recent advances in bioinformatics and biotechnology have extended and expanding the insights of analyzing the segmented genome of the flu virus and exploring the influenza biology [2]. Influenza virus has the following features: (1) it is a single RNA-stranded enveloped virus, (2) its genome is segmented, and it has eight segments, each segment can be encoded to one or two proteins, (3) it is a negative-sense virus, and (4) it can be rated as highly-mutated genome [4]. The virus can infect various hosts, and it has different types and subtypes. The subtypes can be identified according to its surface proteins, haemagglutinin (HA) and neuraminidase (NA) [2,3]. Now, there are 18 HA and 11 NA distinct surface proteins [3,4]. The source of genetic variation is two processes: (1) antigenic drift, or (2) antigenic shift.

As defined by Daoud’s study [1,2], a segmented genome of influenza virus is a composite data point. A composite data point is a dataset from unknow or a well know probability distribution. In machine learning and data mining there are many algorithms that they can be used to analyze, visualize, classify and cluster data points. Usually regular data points, for example, data vectors, univariate data points, and sequences. Processing composite data points is another complicated computational task for existing computational pipelines. Building a statistical learning computational pipeline has several computational challenges [5]. As defined in James et al. [6], statistical learning is a set of unsupervised and supervised computational algorithms that can be used in processing datapoints to extract knowledge and deep understanding about the relationship and structure of data. In other words, statistical learning focuses on learning the relationship and structure from data vectors (i.e., observations of a feature vector). In deep statistical learning, we learn about the relationship and structure of data from distance data vectors after mapping datapoints into different feature spaces using the extension principle of data life cycle [1]. Developing a statistical learning computational pipeline for analyzing the segmented genomes of flu virus is a completed task. One of the computational aspects in statistical learning is to analyze the distance distribution paradigm for the datapoints under consideration [6-8]. A distance distribution paradigm is defined as the probability distribution of a distance measure or metric [6]. In other words, the distance measure or metric is defined as a random variable or random vector [6-8]. In the next section, we shall present a note on the distance distribution paradigm for Mosaab-metric space.

## Technical Implementation

**Fig. 1.**
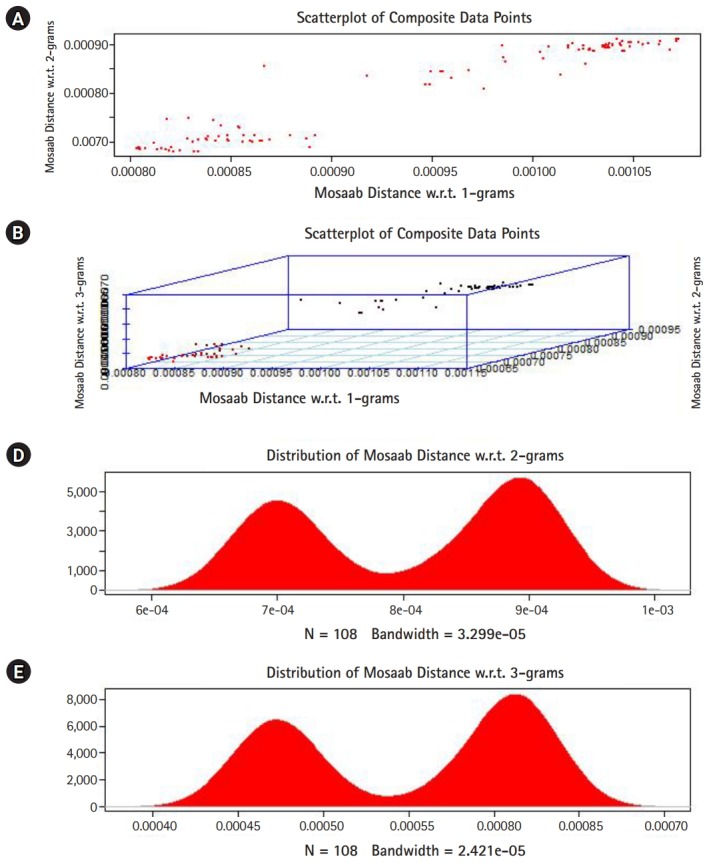
The distance distribution paradigm for Mosaab-metric using 1, 2, and 3 grams feature extraction techniques to analyze composite data points in high dimensional feature spaces (case: 60 composite data points represent two classes of influenza virus, class A and class B). (A) Scatter-plot of composite data points in 2-dimensional space. (B) Scatter-plot of composite data points in 3-dimensional space. (C) The distance distribution paradigm for 1-grams feature space. (D) The distance distribution paradigm for 2-grams feature space. (E) The distance distribution paradigm for 3-grams feature space.

**Fig. 2.**
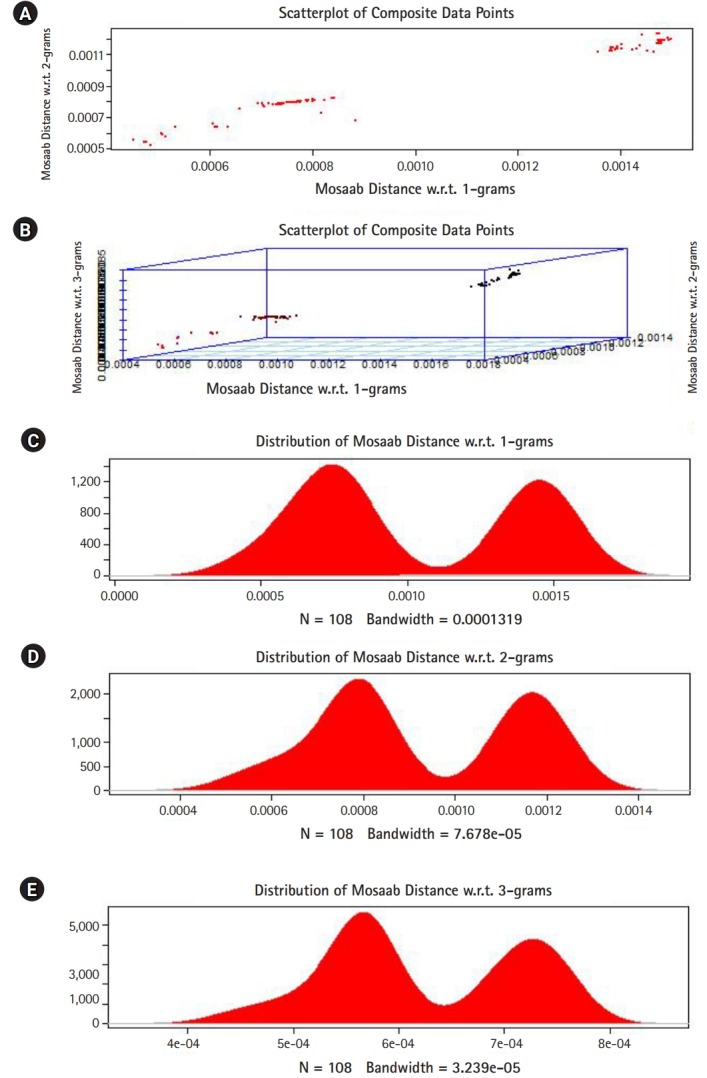
The distance distribution paradigm for Mosaab-metric using 1, 2, and 3 grams feature extraction techniques to analyze composite data points in high dimensional feature spaces (case: 30 composite data points represent one class of influenza virus, class A). (A) Scatter-plot of composite data points in 2-dimensional space. (B) Scatter-plot of composite data points in 3-dimensional space. (C) The distance distribution paradigm for 1-grams feature space. (D) The distance distribution paradigm for 2-grams feature space. (E) The distance distribution paradigm for 3-grams feature space.

**Fig. 3.**
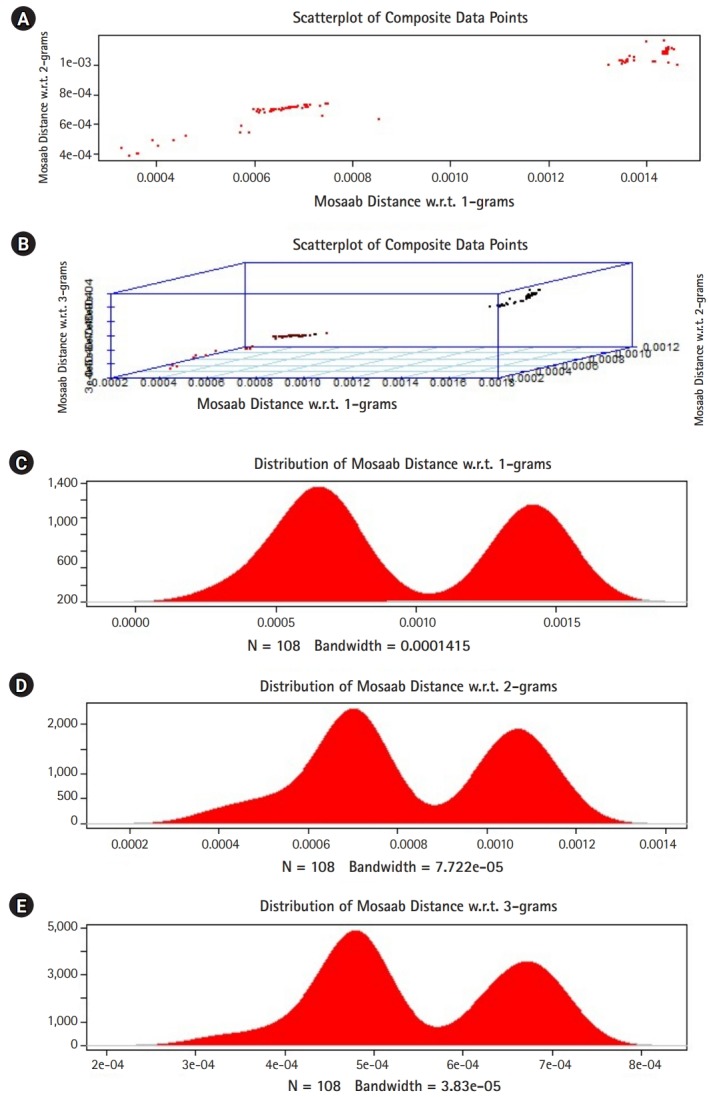
The distance distribution paradigm for Mosaab-metric using 1, 2, and 3 grams feature extraction techniques to analyze composite data points in high dimensional feature spaces (case: 10 composite data points represent one class of influenza virus, class A). (A) Scatter-plot of composite data points in 2-dimensional space. (B) Scatter-plot of composite data points in 3-dimensional space. (C) The distance distribution paradigm for 1-grams feature space. (D) The distance distribution paradigm for 2-grams feature space. (E) The distance distribution paradigm for 3-grams feature space.

In this section, we shall present technical analysis of the deep distance distribution for Mosaab-metric to process segmented genomes of flu virus as composite datapoints, and by using the following three feature spaces: 1-grams, 2-grams, and 3-grams. Mapping each composite data point into various feature spaces by using n-grams technique (in this case n = 1, 2, and 3) has the following outcomes: data-vectors are embedded into feature spaces. The feature spaces are high dimensional spaces. Each composite data point is represented by a dataset, and each dataset is a set of data-vectors. Transforming each set of data-vectors to variance-covariance structure is another information structure, and the outcomes are matrices. Finding the distance between each matrix in the testing dataset and each matrix in the training dataset has the following outcomes: distance values. By using the extension principle of the data life cycle, and in this case by consider three feature spaces (deep statistical learning), the combined outcomes are (3 × 1) distance-data vectors. The distance-data vectors represent a random vector. The random vector has a probability distribution, and since the extracted information is a combination of three feature spaces, then the probability distribution is called the deep distance distribution (or the deep distance paradigm). Now, we shall consider three technical cases about this implementation. We have downloaded 30 segmented genome of influenza virus A, 30 segmented genome of influenza virus B from NCBI-Influenza Virus Database as training datasets [9]. In addition, we have downloaded 108 segmented genome of influenza virus A and B from NCBI-Influenza Virus Database as testing dataset [9]. In case 1, the sizes of training datasets are: 30 segmented genomes for flu A virus, and 30 segmented genomes for flu B virus respectively. The size of testing dataset is 108 segmented genome of influenza virus A and B. Fig. 1 illustrates the analytics of deep statistical learning approach in dealing with composite data points. The first subfigure has two aspects (Fig. 1A) : combining two feature spaces (1 and 2 grams feature vectors) to produce two distance values (or (2×1) distance vector) with respect to a training dataset using the extension principle. In other words, the concept of deep statistical learning is based on extension of the data life cycle. The second subfigure has the same pervious aspects, and by combining three feature spaces, therefore, the result is a (3×1) distance vector (Fig. 1B) . It should be noted that a distance vector is a random vector and it has observations, and those observations are called distance-data vectors. For each feature space, the distance vector as a random vector has a probability distribution, and in this case, it is called the distance distribution paradigm. The distance distribution paradigm for 1-grams, 2-grams, 3-grams feature spaces are illustrated in Fig. 1C, 1D, and 1E , respectively. From these subfigures we can conclude the following: each subfigure has two peaks, each peak represents a class, influenza A virus and influenza B virus. One bell-shaped density curve skewed to the right and another curve skewed to the left. One class has more dispersion than the other, which is in this case influenza A virus. Now consider a training dataset with lack of diversity. Suppose we have two training datasets that represent only one class (in this case influenza A virus), one has 30 composite datapoints and another one has 10 composite datapoints, hence, Figs. 2 and 3 represent the outcomes from these two experiments respectively. Based on the subfigures of Figs. 2 and 3, we have different dispersion maps, two classes, and two peaks. This note has effective conclusions about the impact of size and diversity of datasets on classification results using the distance distribution paradigm.

In this section we presented the technical notes about the distance distribution paradigm for Mosaab-metric using 1, 2, and 3 grams feature extraction techniques to analyze composite data points in high dimensional feature spaces. In the next section we shall present the conclusions.

## Conclusions

**Fig. 4.**
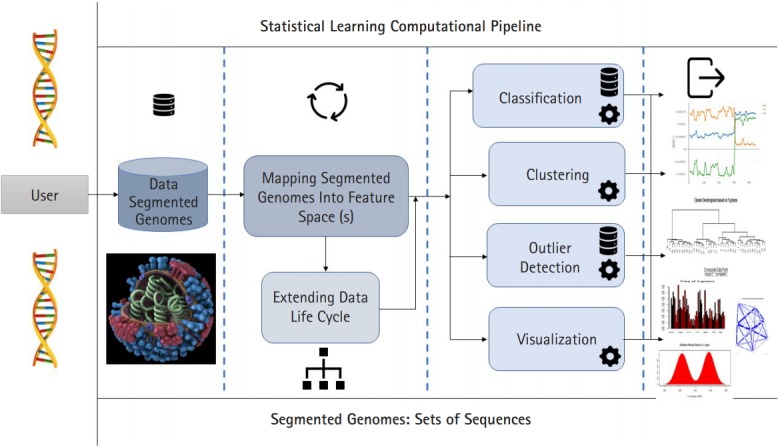
The proposed Statistical Learning computational pipeline to analytically process segmented genomes of influenza virus as composite datapoints (Image-Flu: https://www.cdc.gov/flu/resource-center/freeresources/graphics/images.htm [10]).

In this paper we presented the distance distribution paradigm for Mosaab-metric using three feature spaces: 1-grams, 2-grams, and 3-grams. We technically showed the impact of the size and diversity of training dataset on the classification results. We successfully analyzed the distance distribution of Mosaab-metric space as the most recent metric space in statistical learning research field. This part of analytics (as analytical techniques) about the distance distribution and the dispersion maps is expected to be in a integrated statistical learning computational pipeline for processing and analyzing composite data points (in this case segmented genome of influenza virus, see Fig. 4). The pipeline is sequentially partitioned into components. The first component is to map the segmented genomes into feature spaces (parallel computational mode can be applied), the second component can be executed in parallel mode, and it has different tools (algorithms/techniques). These tools can be summarized as: classification, clustering, outlier detection, and visualization. In the future work, we shall discuss, and present other computational algorithms and/or tools that will be included in this integrated pipeline.
